# A case study on TBM cutterhead temperature monitoring and mud cake formation discrimination method

**DOI:** 10.1038/s41598-021-99439-x

**Published:** 2021-10-07

**Authors:** Jie Fu, Yimin Xia, Hao Lan, Dun Wu, Laikuang Lin

**Affiliations:** 1 College of Mechanical and Electrical Engineering, Central South University, Changsha, 410083 Hunan People’s Republic of China; 2 State Key Laboratory of High Performance Complex Manufacturing, Central South University, Changsha, 410083 Hunan People’s Republic of China; 3 College of Engineering and Design, Hunan Normal University, Changsha, 410081 People’s Republic of China

**Keywords:** Civil engineering, Mechanical engineering

## Abstract

The mud cake is easily formed during the tunnel boring machine (TBM) excavation in clay soils or rocks containing clay minerals. Mud cake will lead to soil disturbance of tunnel face, clogging cutterhead and even affect the construction efficiency and personnel safety. In this study, a discrimination method of mud cake formation based on cutterhead temperature was proposed. An online monitoring system was designed and installed on the slurry balance TBM. The results show that: (a) the cutterhead temperature data can be reliably detected and transmitted by the system; (b) in a tunneling cycle, the temperature at some positions of the cutterhead will increase first and then decrease; (c) during the field test, the temperature variation is around 2.5 °C under the normal condition, but the temperature variation will increase more than 50 °C due to the mud cake or geological change; (d) compared with the cooling rate, mud cake formation can be accurately discriminated.

## Introduction

**Figure 1:**
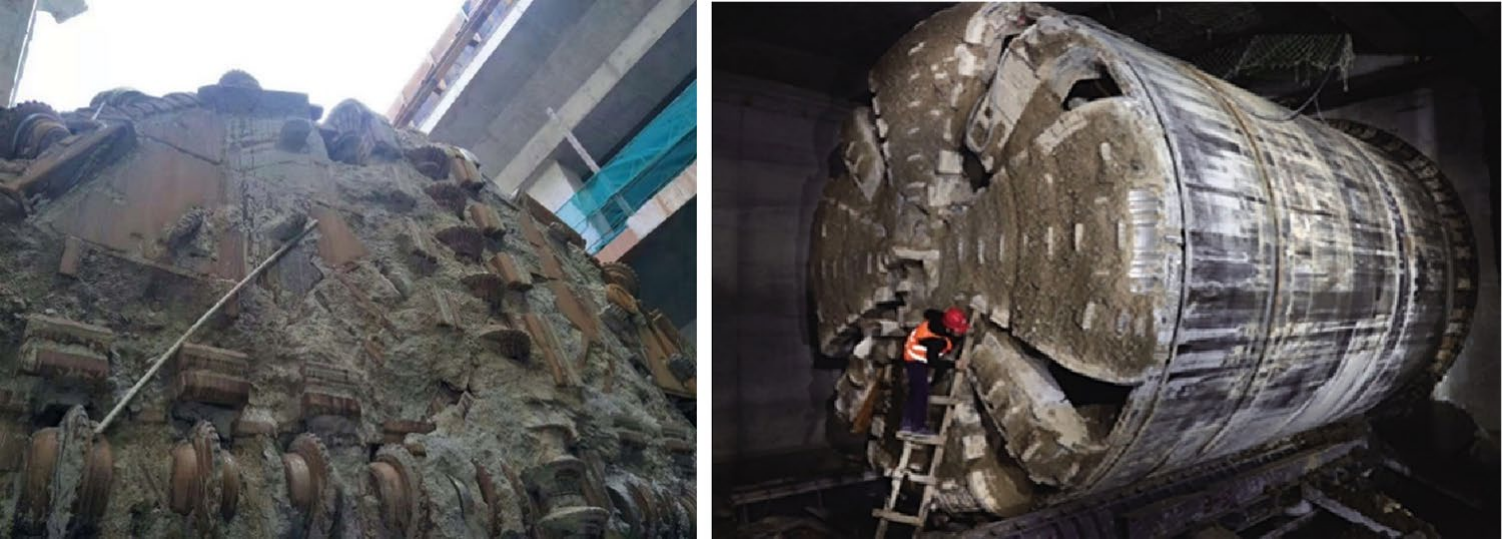
Mud cake at the cutterhead.

Tunnel boring machines (TBMs) have been widely used in urban tunnel projects for roads, subways, and underground pipe corridors due to their high efficiency^1–3^. However, the complex and varied geological conditions could directly affect the tunneling efficiency of TBMs. Earth pressure balance (EPB) TBMs and slurry balance TBMs are mainly used for soft soil. The stability of the cutting surface of these TBMs needs to be ensured by the support fluid. As the viscosity of the support fluid and soil increases, the mud will stick to the surface of the tip and form a mud cake, as shown in Fig. 1. When the mud cake expands, the excavation performance of fluid-supported TBMs will be severely affected, such as the blocked cutterhead and the decreased cutter penetration. The thrust and torque of TBM, as well as the temperature of the cutterhead, will rise sharply^4,5^. In an EPB TBM project of Beijing Metro, the cutterhead temperature has risen more than 60 °C after the formation of mud cake. The high temperature will harden the mud cake and make it difficult to remove^6,7^. If the mud cakes are not eliminated in time, the soil disturbance of the tunnel face will aggravate. That will lead to the collapse of tunnel face, ground subsidence, and other engineering accidents. More seriously, operators who must clean mud cake by entering the cutterhead will face greater safety hazards^8–10^. Therefore, finding and cleaning up mud cake in time will help to improve TBM construction efficiency.

Many scholars started from the cause of clogging and studied the influencing factors in the soft soil layer that easily lead to mud cake or clogging. In particular, the clogging of cutterhead is mainly affected by the open area and the viscous soil material^11^. Hollmann^12^ and Thewes^13^ suggested that the solid rock could also cause the cutterhead clogging. They summarized the effects associated with the clogging tendency of soils and proposed a new test to assess the clogging risk of rocks containing clay minerals. Feinendegen et al.^14^ developed a conical pull-out test to detect (and quantify) the adhesion/clogging tendency of existing rocks or soils in the project's early stages and quantify them as much as possible. Zumsteg et al.^15^ investigated the effects of clay mineralogy and composition of the support fluids on cutterhead clogging and clay paste. A classification scheme for clogging potential based on adhesion parameters' newly defined plugging potential has been proposed^16^. De Oliveira^5^ presented a combined laboratory routine to characterize and evaluate the clogging and fluidity of soils. The above studies analyzed the causes and trends of cutterhead clogging. However, due to the lack of effective monitoring methods, there were still little researches on the mud cake, which forms in the early stage of clogging.

Meanwhile, some methods of reducing or eliminating mud cakes and clogging have been proposed. Milligan et al.^17^ pointed out that increasing the total surface negative charge on solid particles was suitable for viscous soils. Thewes et al.^18,19^ proposed a series of methods to prevent and relieve clogging and applied them to EPB shield TBMs. Zumsteg et al.^20,21^ quantified and explained the limited efficiency of existing conditioning chemicals, by which an enhanced interaction mechanism is also proposed to reduce soil viscosity. Zhang^22^ proposed a block-classification method for assessing block removability and the possibility of cutterhead clogging, which can be used to avoid clogging during TBMs tunneling. Liu et al.^23^ investigated changes of Atterberg limits of clays with dispersant content and their electrochemical mechanism on evaluating the clay conditioning approach for EPB TBM. Due to the complexity and variety of geological conditions, the mud modifier cannot be adapted to all types of geology. Attempting to reduce this risk by adding anti-clogging chemicals could not always produce the desired results. Subsequently, there is an urgent need to monitor the formation of mud cakes in real-time to ensure timely detection and effective treatment.

At present, most of the monitoring methods for TBM cutting temperature were only applicable to laboratory testing. Lv^24^ and Zhang^25^ used the infrared thermal imager to monitor the temperature during cutter and disc cutter cutting tests. Some scholars tried to monitor the temperature of the cutting tools during TBM working. Sahinoglu et al.^26^ used a handy infrared thermometer to monitor the temperature while the TBM was already in operation or when it was stopped to open the excavation chamber cover. This measurement method cannot get the continuous temperature changes during the excavation, while only the temperature value at a certain moment can be obtained. Ji et al.^27^ invited a cutter temperature monitoring system, punching a hole in the cutter for temperature monitoring. However, the temperature sampling interval was relatively long, and the sampling data was inadequate. In addition, more researchers paid attention to the cutting performance of the disc cutters. For instance, the wear and speed of the real-time monitoring device have been successfully tested in the laboratory tests and used in a Chinese TBM project^28–31^. The Mobydic system based on the serpentine manipulator and camera for monitoring TBM cutterhead has been used in Hong Kong^32,33^. In a word, few researchers paid attention to the monitoring of cutterhead temperature, and there was little data to analyze the formation of mud cake by temperature. But the existing monitoring system can be used as the basis for designing the cutterhead temperature monitoring system.

In order to find the mud cake as early as possible, based on the characteristics that the cutterhead temperature will rise during the formation of the mud cake, a mud cake discrimination method is proposed in this paper. In addition, the online monitoring system of the cutterhead temperature was designed as well. The system was applied and verified in the large-diameter slurry balance TBM project. In a word, the online monitoring system could detect the discrimination of mud cake formation without entering the working surface, efficiently improving construction efficiency and saving construction costs and personnel safety.

## Principle of the mud cake discrimination

During TBM tunneling, the cutterhead performs a compound motion of rotation and propulsion through the drive of the main drive and propulsion system. An entire tunneling cycle of TBM tunneling consists of two stages: excavation and erecting segment. The cutterhead and cutters in contact with the soil will raise the temperature of the cutterhead during the excavation phase. When the TBM is erecting segments, the temperature of the cutterhead will gradually decrease because of the natural heat dissipation of the soil or the slurry circulation in the slurry balance TBMs. However, after the mud cake is formed, the temperature of the cutterhead will increase sharply during excavation, which can be regarded as an important indicator to discriminate the mud cake formation.

**Figure 2:**
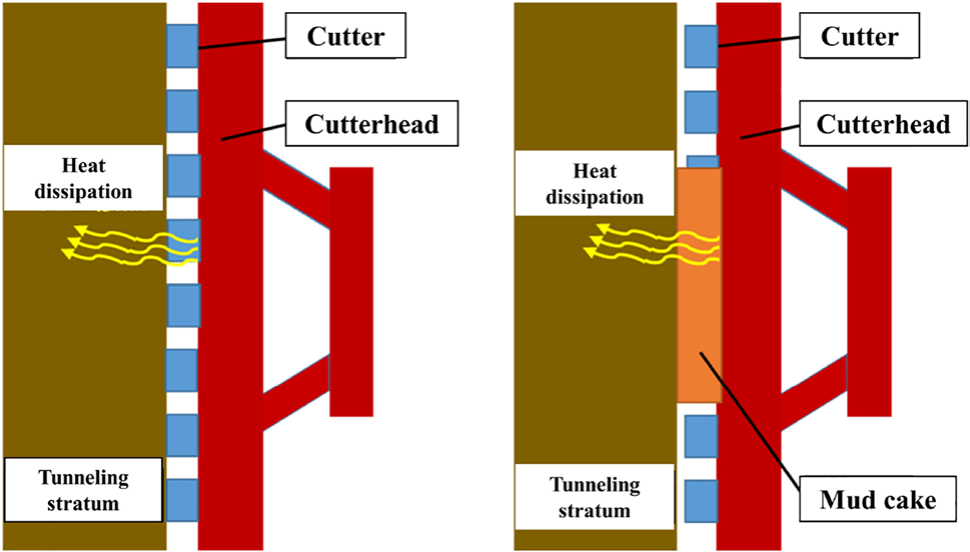
Comparison of heat dissipation of cutterhead before and after mud cake formation (Generated by Microsoft PowerPoints 2016).

If there is a boulder, bedrock protrusions, or stratum changes to harder during the tunneling, the thrust and torque of the TBM will also increase. More heat is released during cutting the rock, and the temperature of the cutterhead suddenly rises. Therefore, the abnormal increase in the temperature of the cutterhead may be due to not only the formation of mud cake but also the geological changes. Focusing only on the maximum temperature value change of the cutterhead does not accurately discriminate whether a mud cake is formed. As shown in Fig. 2, when the mud cake is not formed, the heat generated by friction on the cutterhead surface will directly flow into the tunneling stratum and dissipate. But the mud cake will cover the surface of the cutterhead after it is formed. At this time, the mud cake will separate the cutterhead from the tunneling stratum and slow down the heat dissipation. Therefore, the heat dissipation rate of the cutterhead is another important indicator to judge whether mud cake is formed or not. During the erecting segment stage, the temperature of the cutterhead will decrease because of no friction heat. The temperature cooling rate of the cutterhead in the erecting segment stage is used for distinguishing mud cake formation and geological changes. The following equation can express the cooling rate of the cutterhead.

1 $$k = \left| {\frac{dT}{{dt}}} \right| = \left| {\frac{{T_{0} - T_{1} }}{{t_{0} - t_{1} }}} \right|$$

where *T*_*0*_ represents initial temperature, *T*_*1*_ represents final temperature, *t*_*0*_ represents initial time, *t*_*1*_ represents final time, *k* represents the cooling rate of the cutterhead with the unit of °C/min.

The maximum cutterhead temperature (*T*_*max*_) and the cooling rate (*k*) of each tunneling cycle should be recorded. Mud cake has formed when *T*_*max*_ suddenly increases and *k* gradually decreases. In addition, the type of TBM, the geological condition, and the structure of the cutterhead could all affect the temperature of the cutterhead during the excavation. Therefore, it is necessary to rely on the temperature data collected in the same working conditions for analysis.

## Design and development of the monitoring system

### Development of the monitoring system

**Figure 3:**
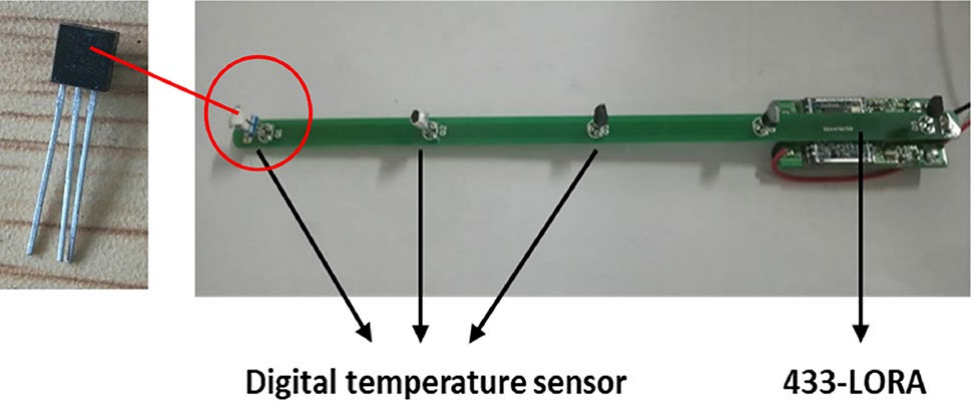
The integrated sensor module.

In this study, 433-LORA wireless communication modules with MSP430 CPU and digital temperature sensors are employed. The whole temperature monitoring system for cutterhead consists of online temperature monitoring sensors, a wireless receiving system, and a laptop with the self-developed software platform. In addition, each sensor has a specific address when transmitting signals. All sensors share a receiving device for signal transmission, and the self-developed software carries out digital signal processing. The online temperature monitoring sensor includes five digital temperature sensors, one 3.8 V battery, and one wireless transmitting module. The sealant waterproofs all components. According to the preliminary investigation of the Hangzhou Wangjiang Road Crossing River Tunnel in China, the temperature of the cutterhead is usually 20–30 °C during the tunneling process. DS18B20 digital temperature sensor with a test range of − 55 °C to 120 °C is applied to the monitoring system and the integrated sensor module is illustrated in Fig. 3.

**Figure 4:**
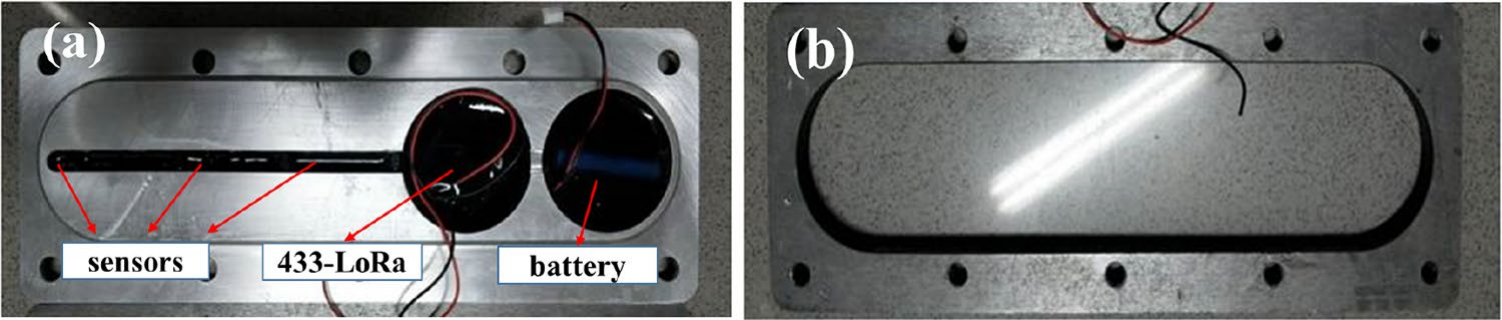
Sensor structure installed in the field: (**a**) Sensor body, (**b**) Sensor base.

The sensor module shown in Fig. 4a is pre-mounted into the designed aluminum alloy plate, ensuring the stability and convenience of the sensor installation. The electronic components are sealed with the sealant during installation to prevent sensor failure in the wet environment. The bosses designed in the module are easy to location during installation and allow the sensor to contact the cutterhead for more accurate temperature measurement. The online temperature monitoring sensor base shown in Fig. 4b is welded to the back of the cutterhead. Next, the sensor body is mounted on the sensor base by bolts and coated with thermal silica gel on the contact surface for heat conduction.

### Design of the monitoring system

To accurately monitor the temperature, the digital temperature sensor is applied to the monitoring system, where the data acquisition device can directly read the temperature data. Since the sensor is mounted on a rotating cutterhead, it is difficult to transmit the signal to the host computer at the rear through the signal line. Moreover, the signal could be easily disturbed and the transmission is unstable when applying a rotary joint as the signal transmission. Nowadays, wireless monitoring has been applied in many projects as an important means of TBM cutterhead inspection^29–31^. Therefore, the wireless transmission technology is applied to the temperature monitoring system for cutterhead to obtain a real-time signal from the temperature sensor in this study. The transmitted temperature data is displayed on a self-developed PC software platform.

**Figure 5:**
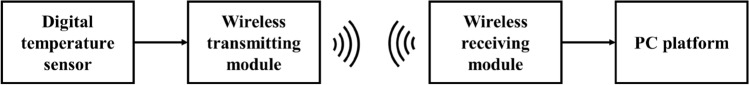
Constitution diagram of the mud cake temperature monitor system for TBM cutterhead.

Moreover, the constitution diagram of the entire monitoring system is shown in Fig. 5. It is also necessary to consider the surrounding environment, signal shielding, and other issues during the actual installation. The sensor structure, the mounting location, and the transmission line layout are designed according to the actual situation on site, respectively.

## Field test

### Project introduction

**Figure 6:**
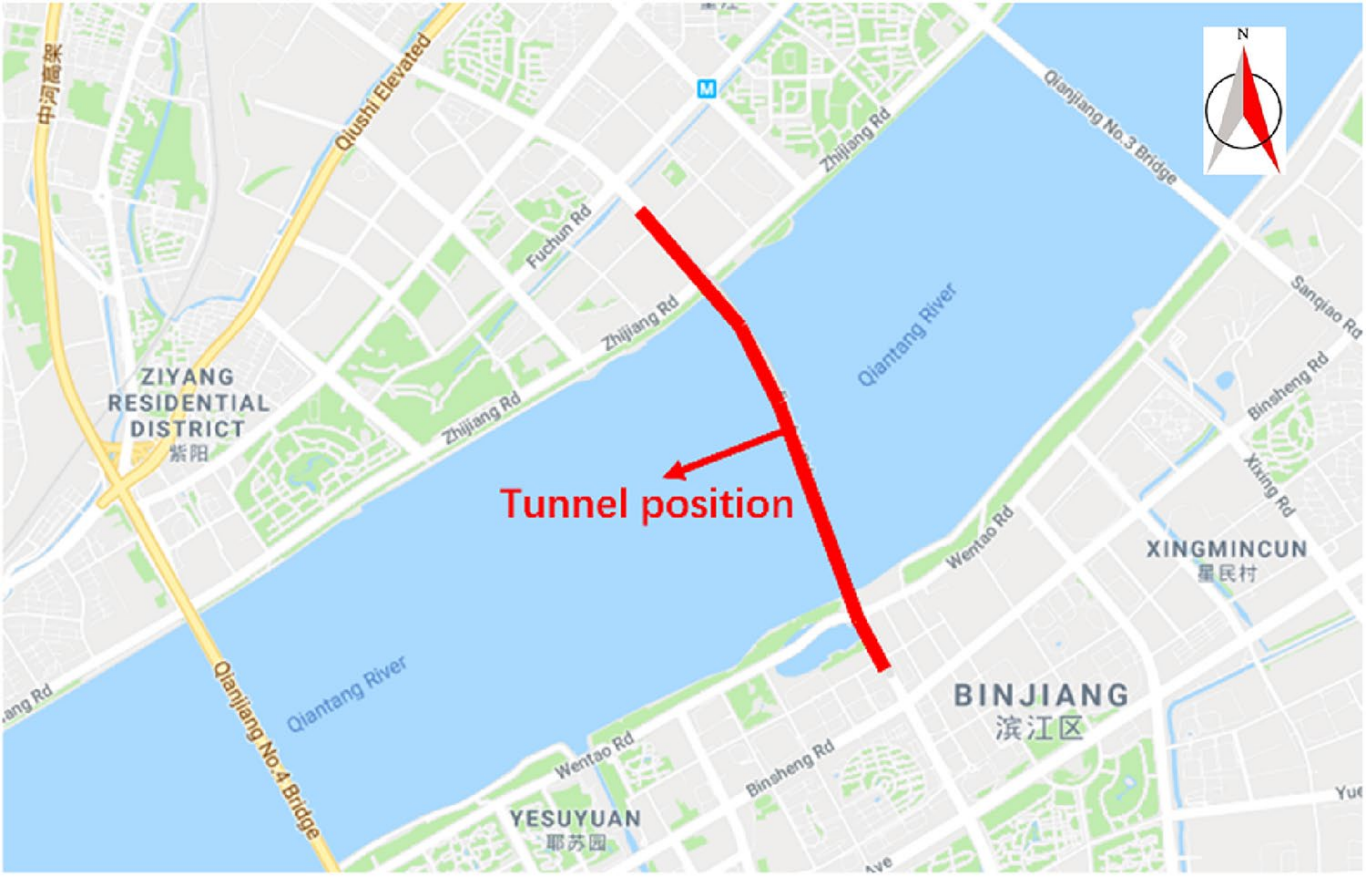
The location of field test (Generated by Baidu map and Microsoft PowerPoints 2016).

The monitoring system was applied in Hangzhou Wangjiang Road Crossing River Tunnel in Zhejiang Province of China. As shown in Fig. 6, the tunnel crosses the Qiantang River from south to north. Specifically, the tunnel is endowed with a length of 1835.84 m, which passes through the silty clay and silty sand stratum. Therefore, the mud cake of the cutterhead might be formed during the excavation process. The construction equipment is a large-diameter slurry balance TBM, and the construction company is China Railway 14th Bureau Group Shield Engineering Co., Ltd. The TBM is equipped with a normal pressure cutterhead which diameter is 11.64 m. The buried depth of the TBM construction section is 19.1–34.2 m. The geological conditions of the construction by TBM are shown in Table 1.

**Table 1:** Geological conditions of excavation strata.

Exploration distance (m)	Excavation geology types
ZK1 + 0 ~ ZK1 + 346	Silty sand, silty clay, silt
ZK1 + 346 ~ ZK1 + 545	Silty clay, silt, sand
ZK1 + 545 ~ ZK1 + 810	Sandy silty clay, silty clay
ZK1 + 810 ~ ZK2 + 005	Mucky clay, silty clay
ZK2 + 005 ~ ZK2 + 335	Silty clay, gravel
ZK2 + 335 ~ ZK2 + 540	Mucky clay, silty clay
ZK2 + 540 ~ ZK2 + 840	Silty sand, silt, silty clay

**Figure 7:**
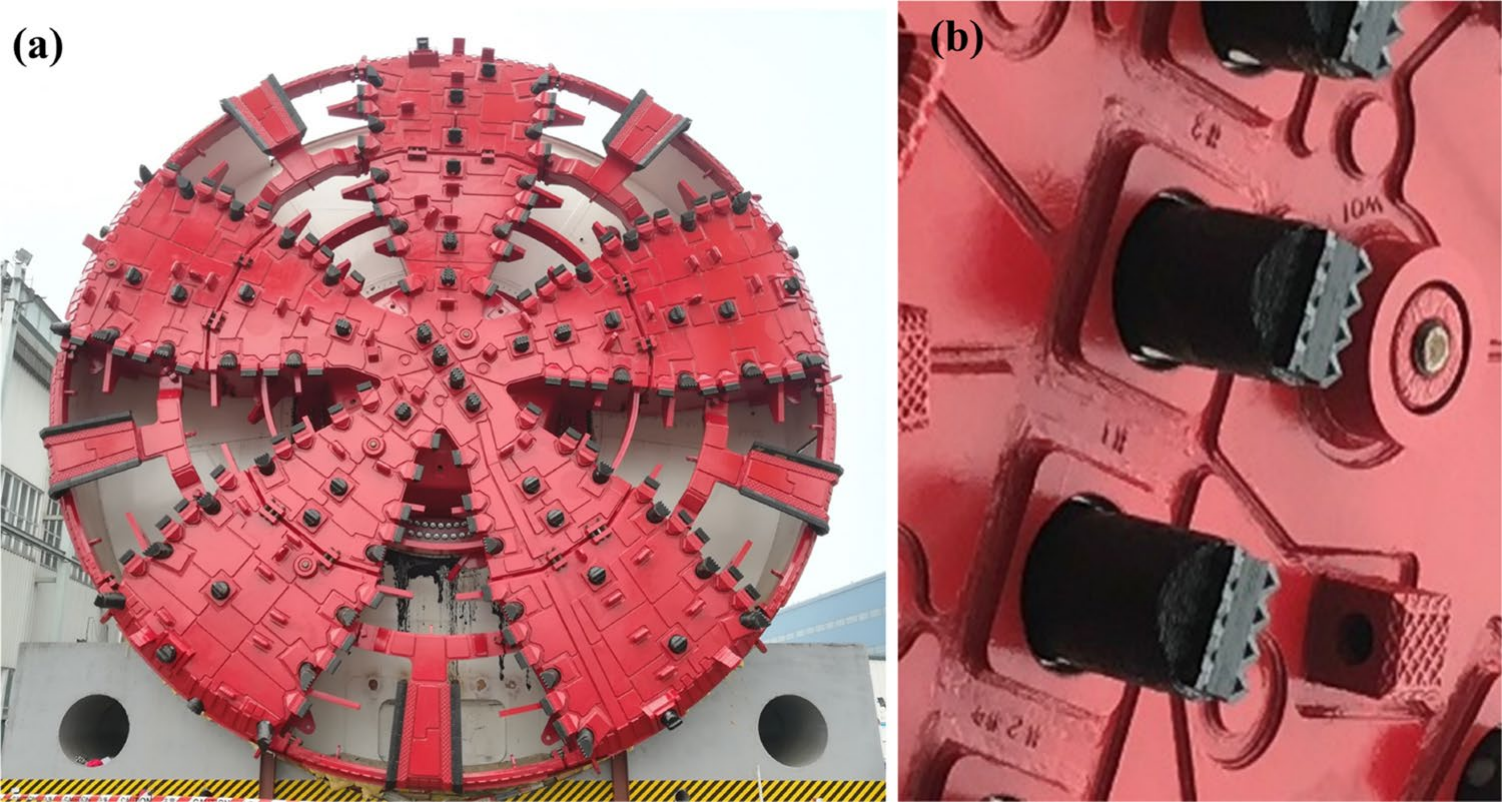
Cutterhead and cutter of the large-diameter slurry balance TBM: (**a**) cutterhead, (**b**) normal pressure replaceable cutters.

Figure 7 shows the cutterhead and cutter of the TBM. In particular, the cutterhead consists of the cutter-changing chamber and one center cone, which can change the cutter under conventional air pressure. Separated by the gates, the interiors of cutterhead are hollow. The operators can enter the cutter-changing chamber and center cone directly. The temperature sensor is mounted on the back of the center cone and detects the real-time temperature. The field installation is designed to validate the reliability of the monitor system and the accuracy of cutterhead temperature during the TBM excavation.

### Installation of the monitoring system

**Figure 8:**
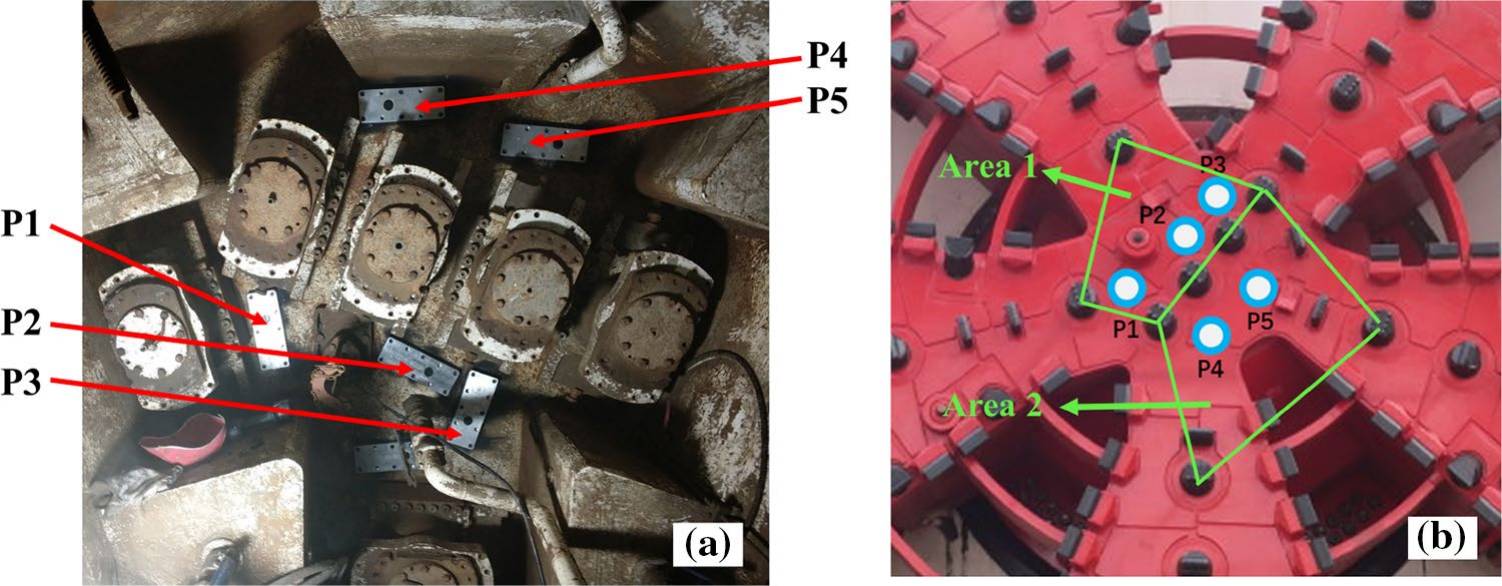
Sensors installed position of the cutterhead: (**a**) reverse side of cutterhead, (**b**) front side of cutterhead.

**Figure 9:**
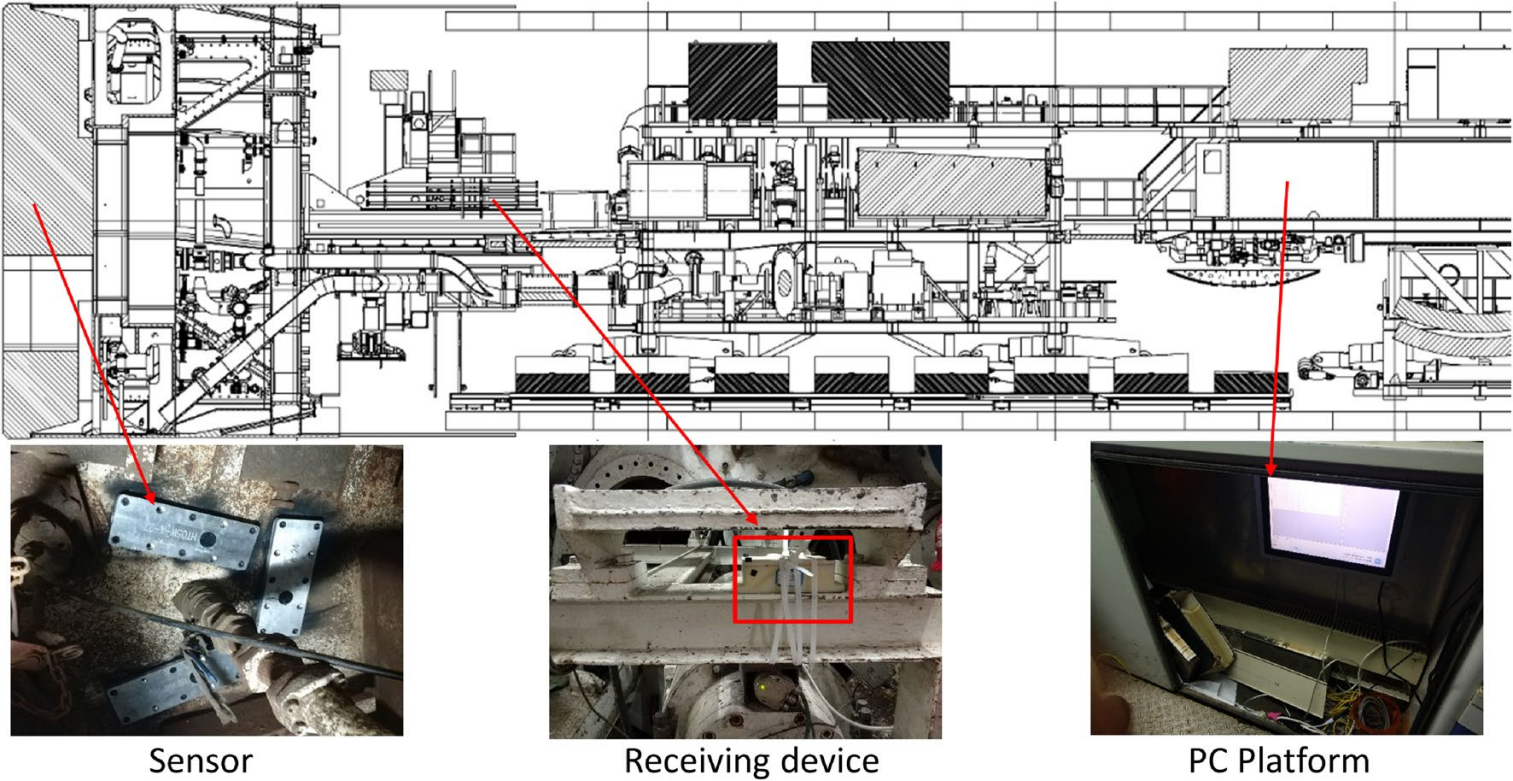
Installation position of monitoring system (Generated by Auto CAD 2010 and Microsoft PowerPoints 2016).

**Figure 10:**
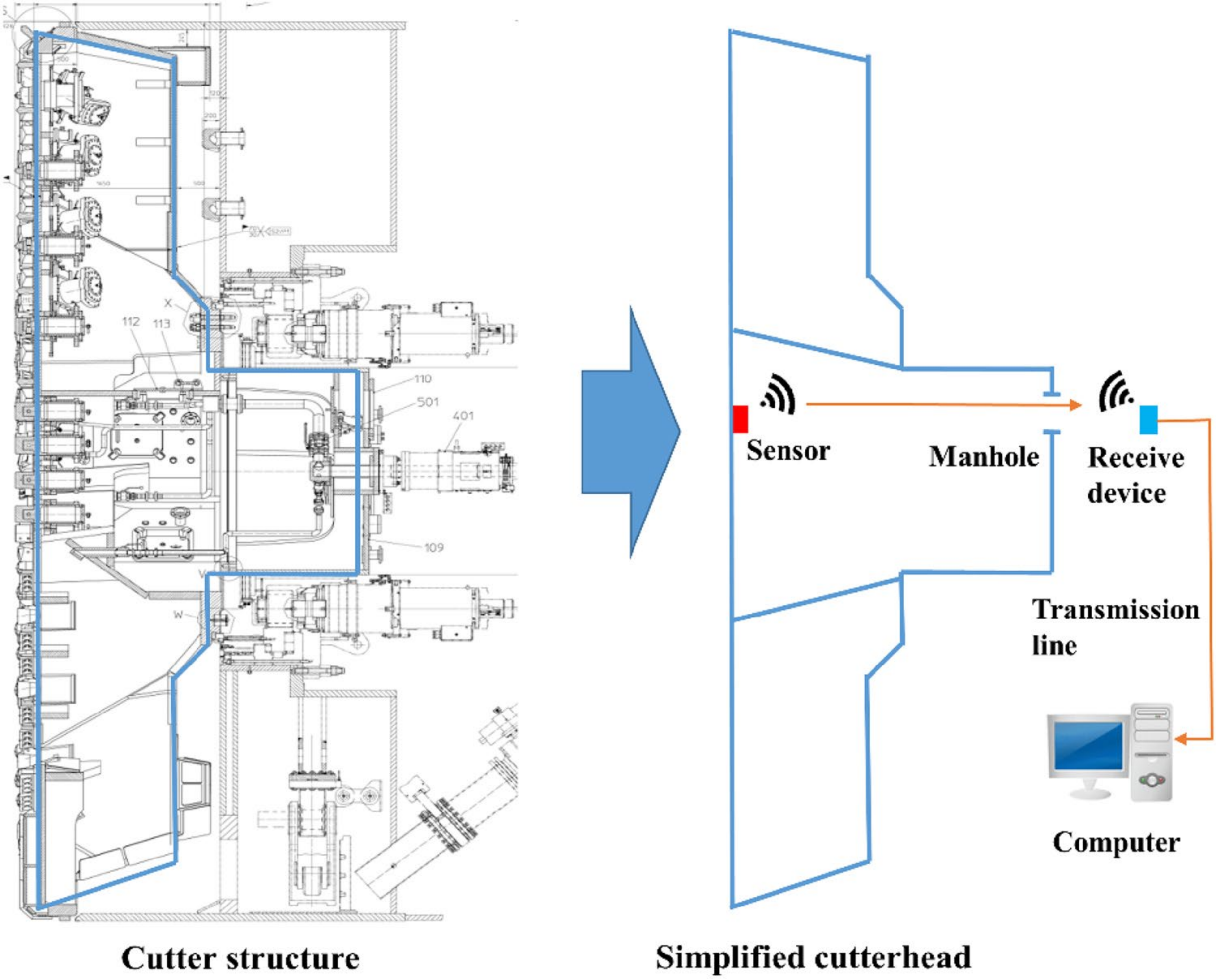
Cutterhead structure and signal transmission diagram (Generated by Auto CAD 2010 and Microsoft PowerPoints 2016).

As shown in Fig. 8, five sensors were mounted in the cutterhead center where mud cakes are most likely to be formed. Among them, the measuring points P1, P2, and P3 are close to the center of the cutterhead. P4, P5 are close to the cutterhead opening. The manhole in the excavation chamber was opened to avoid any screen effect on the wireless signal transmission from the sensor. The receiving module was kept on a transport rack and connected to the monitor laptop installed in the main control room through cables, as shown in Fig. 9. The cutterhead structure and signal transmission diagram are shown in Fig. 10. The signal between the sensor and the receiving device is transmitted wirelessly, while the signal between the receiving device and the computer is transmitted by cable. The orange arrow line shows the signal transmission in Fig. 10. Therefore, the rotation of the cutterhead would not affect the signal transmission.

**Figure 11:**
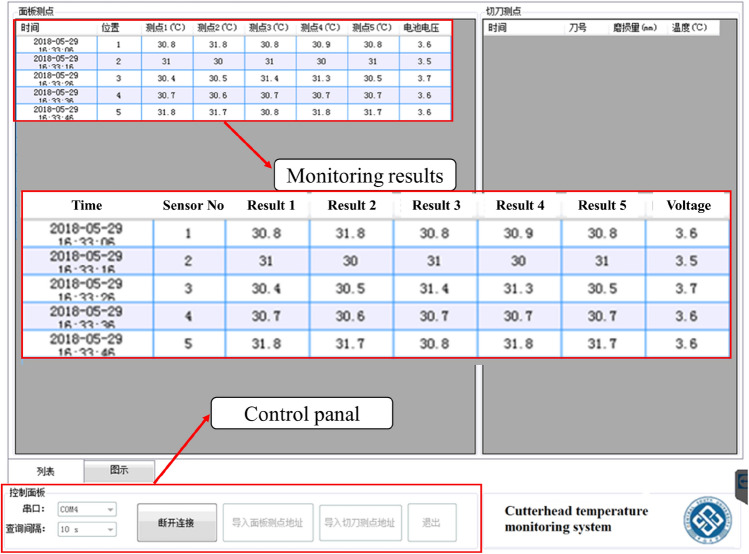
Self-developed temperature monitoring software.

A self-developed software was employed to collect and display the sensing data when the TBM was digging and thrusting. Figure 11 shows the software interface for temperature measurements where seven system parameters (including serial number, temperature value, and device voltage value) are displayed in real-time. To increase the signal-to-noise (SN) level, the average of 5 temperature points measured by each sensor is recorded at one time. In addition to temperature measurements, the real-time system can monitor the power of each sensor to avoid battery exhaustion.

### Temperature data analysis

The temperature monitoring system of cutterhead was tested in the slurry balance TBM construction site in Hangzhou from April to June 2018. After installation of the entire monitoring system, the real-time temperature change of the cutterhead could be detected with good stability by the monitoring system.

#### Single cycle temperature data analysis

**Figure 12:**
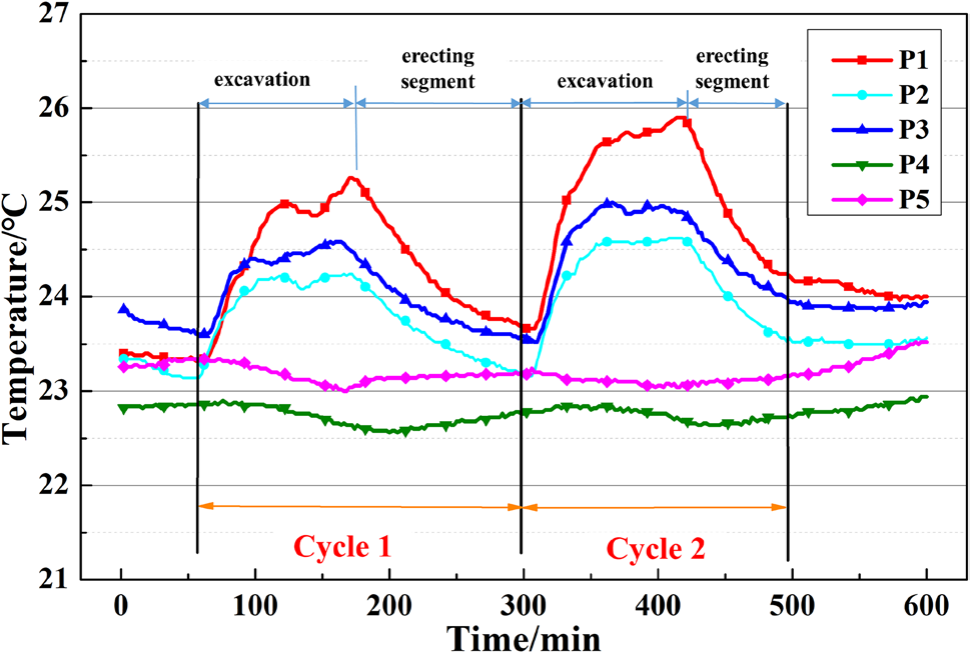
The temperature of cutterhead on April 10, 2018.

On 10th April, the slurry balance TBM was achieved for tunneling two cycles with average thrusts of 52,537 kN and 55,133 kN. The torque for each cycle was 1896 kN·m and 1760 kN·m, respectively. As illustrated in Fig. 12, the temperatures of the five test points ranged from 22.5 to 24 °C, which was close to the temperature of the tunnel. The temperature change of the cutterhead was within 3 °C. Furthermore, temperatures of measuring points (P1, P2, P3) went up rapidly as the TBM started tunneling. After around 50 min, the temperatures in Cycle 1 and Cycle 2 reached 25.26 °C and 25.9 °C, respectively. When the excavation process was completed, and the erecting segment stage began, the temperatures of the three test points decreased gradually.

The temperature rising at P1, P2, and P3 is caused by the heat derived from the friction between the cutter and soil when the cutterhead is rotating. The working temperature of the excavation stage would be 25–26 °C, as shown in Fig. 12. After excavation, the mud water circulation system transports muck produced by the excavation through pipes, leading to heat dissipation and temperature decrease. On the other hand, the temperature of P4 and P5 did not change significantly. The difference in temperature change between P1 (or P2, P3) and P4 (P5) is heat dissipation. The temperature change of cutterhead is affected by many factors, including the heat produced by excavation, heat dissipation caused by slurry flow, and ambient temperature. The slurry between the cutterhead and cutting face is squeezed into the slurry warehouse through the opening of the cutterhead. Therefore, the better fluidity of the slurry would lead to a lower temperature at the cutting surface. The temperature of the cutterhead might not change or even decrease during excavation. As shown in Fig. 8, the cutters around P1, P2, and P3 easily form a closed area when excavation. It will cause slower heat dissipation of the cutterhead in this area. However, a cutterhead opening in the closed area is formed by the cutter around P4 and P5, contributing to a high heat dissipation rate. Hence the temperature change is not obvious, or even the opposite change law.

#### Long-time continuous data analysis

Long-time continuous testing of the cutterhead temperature monitor system had been carried out from April 9th to April 19th. The stratum of the slurry balance TBM was loose silty soil and sand layer. The thrust was between 52,000 kN and 57,000 kN, and the torque fluctuated around 2000 kN·m. The average tunneling speed was 23 mm/min. During this time, there was no obvious change in the stratum and other excavation parameters.

**Figure 13:**
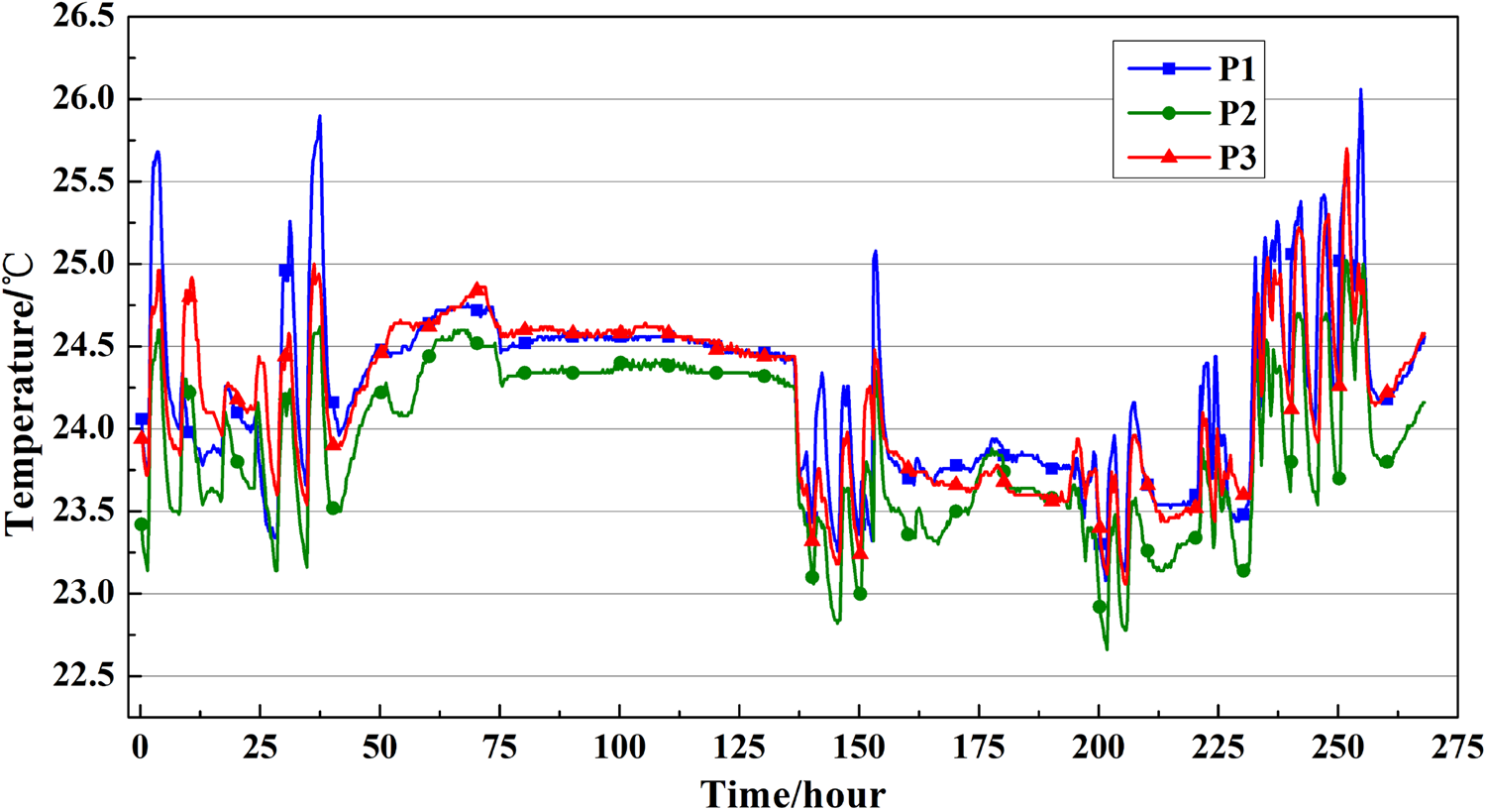
P1 P2 and P3 temperature data from April 9th to April 19th.

As shown in Fig. 13, the P1 P2 and P3 temperatures changed significantly for each excavating cycle, while the temperature was almost stable for the TBM, which is at idle. In the long-time continuous testing of the temperature monitor system, each distinct peak in the temperature change corresponds to a transition of TBM from the excavation stage to the erecting segment stage. While during the continuous tunneling, the maximum temperature of the measuring point gradually rose and reached a maximum value of around 26 °C. In 0–50 h, the temperature change of the test point is relatively normal, which is due to the long interval between each tunneling cycle. The temperature will gradually rise at the tunneling stage and gradually drop to ambient temperature at the erecting segment stage. In 75–125 h, the temperature of the cutterhead was almost stable due to the TBM being overhauled. The temperature curves increase sharply and change faster in 225–250 h. During this period, the excavation speed of the TBM is accelerated, the tunneling cycle becomes shorter, and the cutterhead temperature enters the next tunneling cycle before it drops to the ambient temperature, which causes the increase in temperature rise.

In general, during this period, the temperature data transmission is continuous and stable. The monitoring system is not affected by the surrounding environment and can be reliably used for long-term temperature monitoring with detecting time of at least 10 days. Due to the normal state of excavation, the temperature rise of the measuring point during excavation is only 1.5–2 °C. The long-term test has further verified the reliability of the temperature monitoring system.

## Discussion of abnormal temperature rise

**Figure 14:**
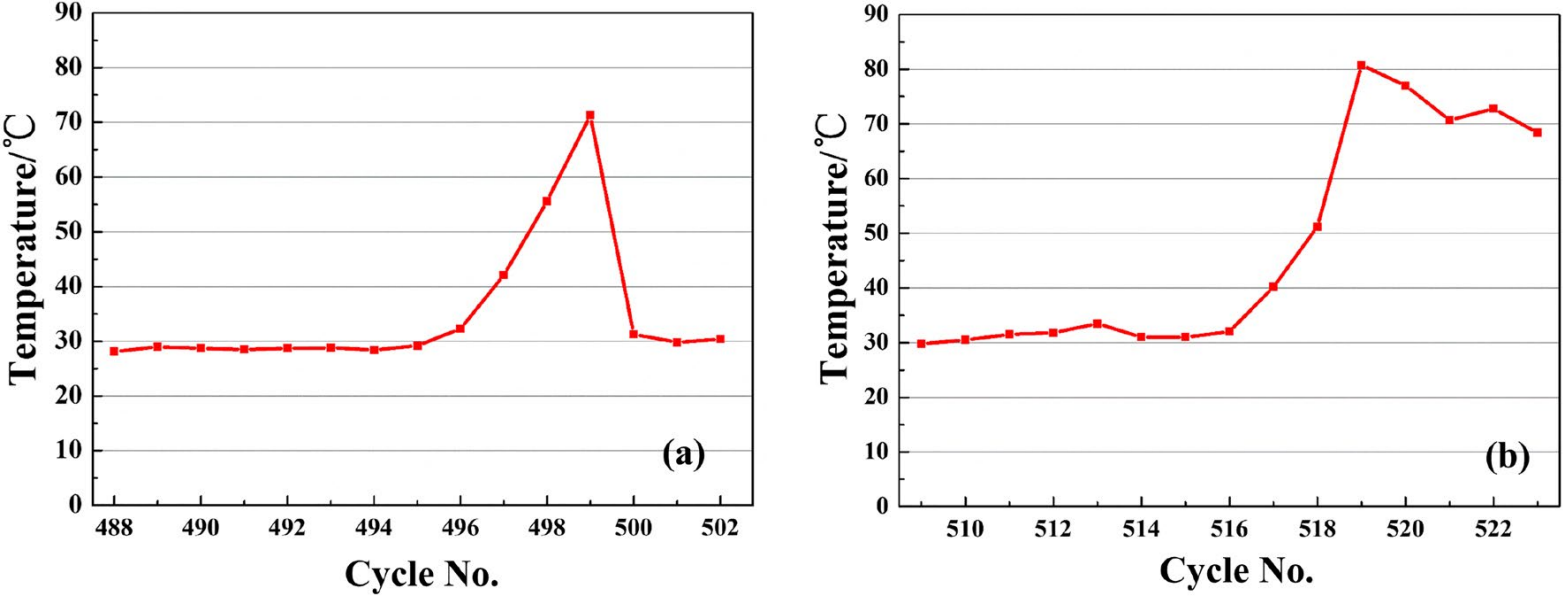
Temperature maximum of P1: (**a**) first, (**b**) second.

**Figure 15:**
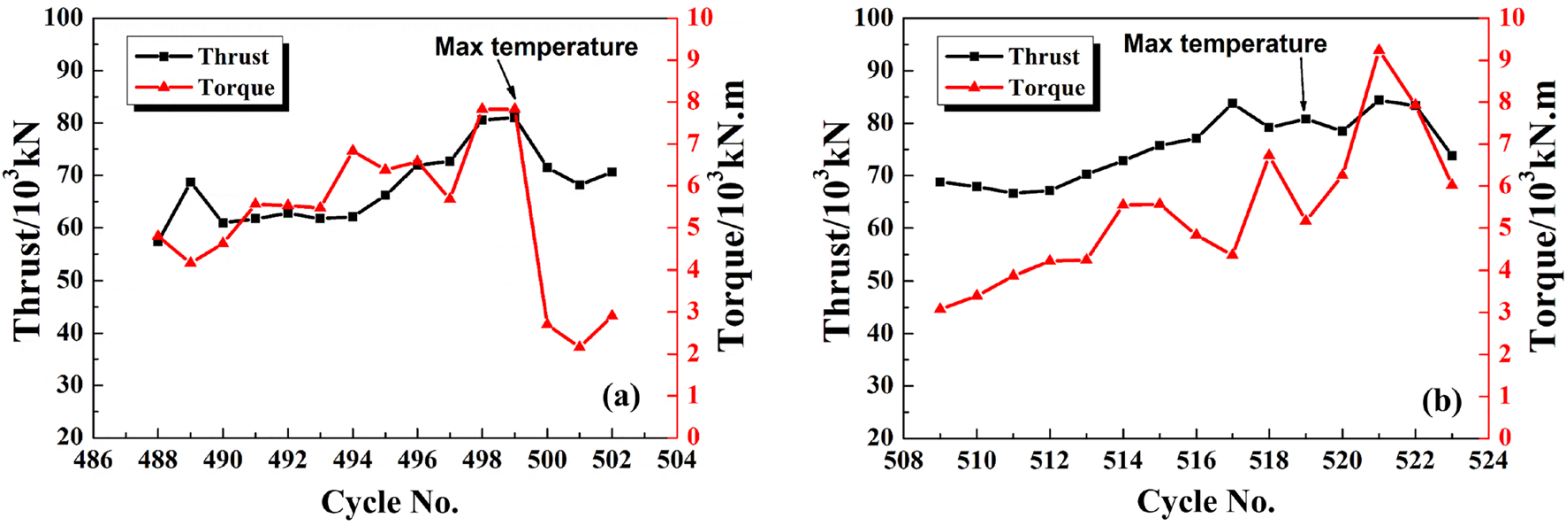
Average thrust and torque: (**a**) first, (**b**) second.

In the entire temperature monitoring process, we noticed two abnormal rising trends of the cutterhead temperature. These two abnormal temperature changes of P1 were shown in Fig. 14. The temperature went up to about 75 °C which was twice as those of the normal tunneling cycles. Since the sensor was mounted on the backside of the cutterhead, it was conceivable that the temperature of the front of cutterhead will be higher. Figure 15 illustrated the trend of the average thrust and torque of several tunneling cycles when two temperatures rose abnormally. The thrust of the slurry balance TBM rose to 80,000 kN, and the torque reached 7000 kN·m simultaneously. When these tunneling parameters rose significantly, excavation speed slowed down. Therefore, it can be discriminated that the excavation stage was under an abnormal circumstance. To give a more specific clarification, further analysis of the cooling rate would be required when the slurry balance TBM shuts down.

**Figure 16:**
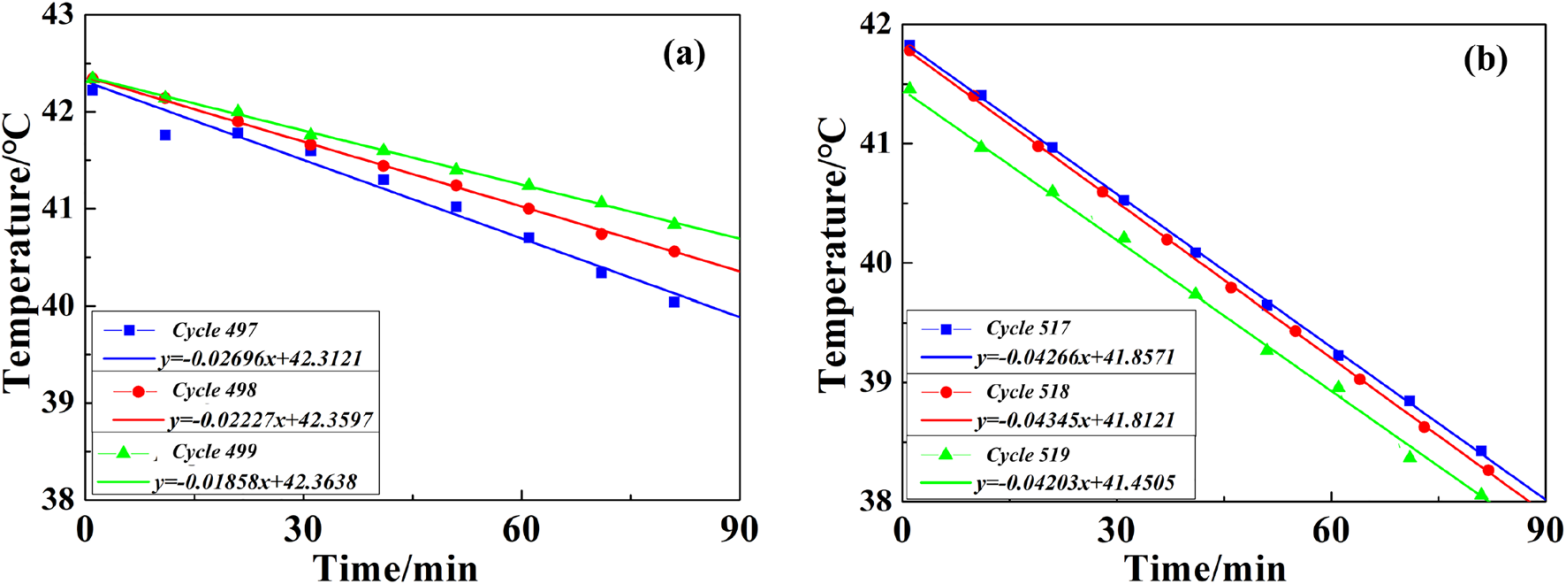
Temperature change regression curve: (**a**) first, (**b**) second.

**Figure 17:**
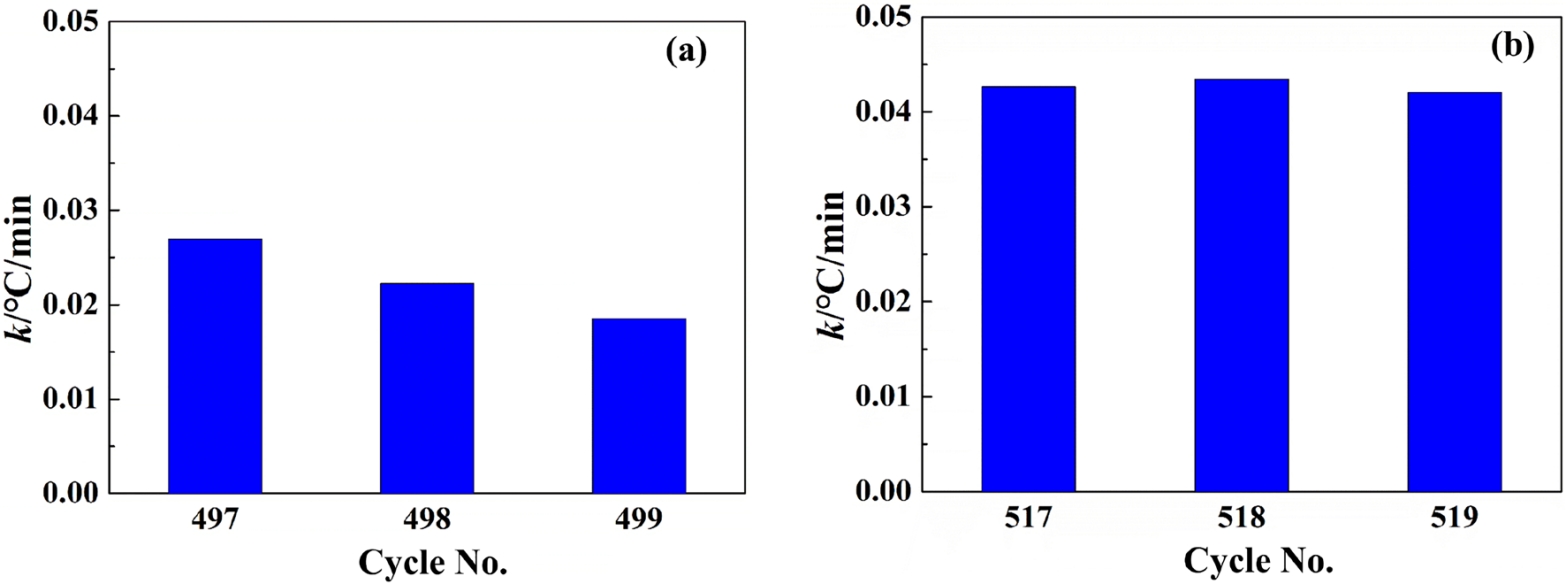
Cooling rate: (**a**) first, (**b**) second.

The heat transfer rate between two objects is positively correlated with their temperature difference, i.e., the greater temperature difference between two objects would lead to faster heat transfer from the high-temperature object to the low-temperature object. Therefore, we choose the same initial temperature to analyze the cutterhead cooling process, or the temperature difference will impact the cooling rate. We selected the P1 measuring point to analyze the temperature variation at the tunneling cycle with the highest temperature and the previous two tunneling cycles. The cycle number of the first part is 497–499, and the second part is 517–519. The initial temperature of the analysis is around 42 °C with a cooling time of 90 min. Cooling rate k (Formula 1) is obtained by regression analysis. The regression curve was shown in Fig. 16. The temperature of the first abnormal situation dropped by 1.5–2.5 °C in 90 min, and the cooling rate k was below 0.03 °C/min with the minimum value of only 0.0186 °C/min. For the second time, the temperature dropped by nearly 4 °C. The average value of k was about 0.0427 °C/min. The first cooling rate was significantly lower than the second one. As shown in Fig. 17a, the k of the three tunneling cycles showed a significant downward trend for the first time. It was very likely that the mud cake was gradually developing during the excavation, and the increase in thickness would lead to more difficult heat dissipation. As shown in Fig. 17b, three curves were almost parallel. The cooling rate of the 517–519 cycle does not show a downward trend and is significantly higher than the 497–499 cycles. Based on the above phenomenon, we judged that in 497–499 tunneling cycles, the mud cake had formed at the cutterhead.

**Figure 18:**
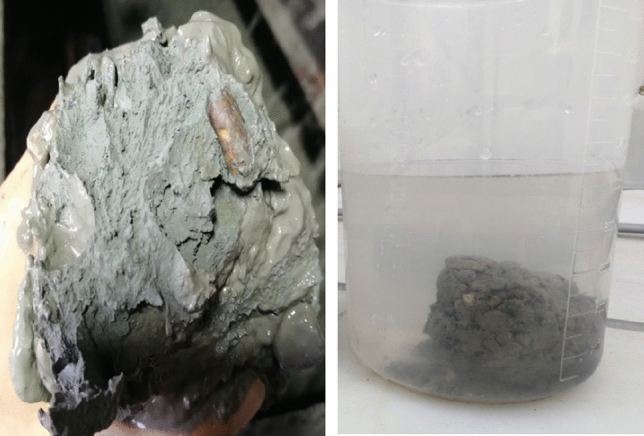
Mud cake sample was taken out form cutterhead.

When the temperature appears abnormal for the first time, the mud cake forming was confirmed by entering the cutting face and checking. The mud piece taken out from the cutterhead was shown in Fig. 18. It was a hard mud formed by the cementing of clay and gravel, which is indiscerptible in water. During the construction process, 1 ton of dispersant was injected into the cutterhead. To eliminate mud cakes, we used the dispersing agent and rotated the cutterhead simultaneously. After that, the thrust and torque of TBM, as shown in Fig. 14, in the next cycle was significantly reduced, and the TBM returned to normal working when mud cake was cleaned. However, at the second time of the abnormal temperature, the addition of dispersant in front of the cutterhead failed to return the tunneling parameters and temperature normal. After checking the cutterhead, no mud cake was found. The TBM advanced 2 m per excavation cycle. The excavation distance during the second temperature abnormality was ZK2 + 038 m. As shown in Table 1, the excavation geology contained gravel. The strata containing gravel is harder than Silty clay, and the load will increase when excavation in gravel strata, resulting in increased heat production. That is the main reason for the increase in excavation parameters and temperature. Therefore, the geological change resulted in the temperature rose abnormally, while the cutterhead cooling rate did not change. In a word, by comparing the cooling rate, the mud cake formation and geological changes can be distinguished more accurately.

## Conclusion

In this study, the discrimination method of cutterhead mud cake formation based on temperature is proposed, and a monitoring system for cutterhead temperature is developed. The monitoring system has been applied to the project at the Hangzhou Wangjiang Road Crossing River Tunnel in China. Based on the test result, the conclusions are:

1. The monitoring system uses a digital temperature sensor and a 433-LoRa wireless transmission module to achieve stable signal transmission under the condition of the cutter head rotating. The results of the monitoring data show that the monitoring system has high reliability. The self-developed software can store the monitoring data in real-time.
2. The cutterhead temperature monitoring data shows several characteristics: (a) The cutterhead temperature will rise and fall during each tunneling cycle. Under normal circumstances, the temperature difference is generally around 2.5 °C. (b) When the TBM continues to excavate, the maximum temperature of cutterhead could continuously rise. (c) When the geological hardening or mud cake formation has occurred, the cutterhead temperature rose abnormally. The maximum temperature reached above 70 °C, which was 50 °C higher than that in a normal case.
3. The discrimination method of cutterhead mud cake has been verified at the construction site. The cooling rate was 0.04272 °C/min when the stratum became hard. The cooling rate of per-cycle decrease when the mud cake had formed, and the minimum value was 0.01862 °C/min. The discrimination method of mud cake through the change of cutterhead temperature includes the following two factors. The abnormal rise of maximum temperature at the cutterhead during the excavation stage. The cooling rate reduced during the erecting segment stage.

The monitoring system has been applied in the large diameter slurry balance TBM and has the potential to be extended to EPB TBMs. By placing sensors in more locations, the entire cutterhead mud cake formation can be monitored. In the future, temperature data will be used as another important excavation parameter while evaluating the cutting performance of the TBMs.
